# Biological Oriented Immediate Loading: A New Mathematical Implant Vertical Insertion Protocol, Five-Year Follow-Up Study

**DOI:** 10.3390/ma14020387

**Published:** 2021-01-14

**Authors:** Fabrizio Bambini, Giulia Orilisi, Alessandro Quaranta, Lucia Memè

**Affiliations:** 1 Department of Clinical Sciences and Stomatology, Polytechnic University of Marche, Via Tronto 10, 60126 Ancona, Italy; f.bambini@staff.univpm.it (F.B.); lucia.meme@hotmail.it (L.M.); 2 School of Dentistry and Oral Health, Griffith University, Brisbane, QLD 4101, Australia; profalexquaranta@gmail.com

**Keywords:** dental implants, marginal bone loss, soft tissue, immediate dental implant loading, oral bacteria

## Abstract

One of the current major challenges in implant therapy is to minimize marginal bone loss around implants, since it can trigger bacterial colonization of the implant’s neck, leading to its failure. The present study aimed (1) to scientifically validate a new mathematical rule based on soft tissues thickness, for choosing the correct implant position with respect to the bone level, in order to provide a better tissue adaptation to the abutment/implant surface to avoid bacterial invasion, and (2) to apply this mathematical rule to the Biological Oriented Immediate Loading (B.O.I.L.) surgical protocol, avoiding peri-implant bone resorption. N. 127 implants were inserted following B.O.I.L. protocol: implants were placed according to the mathematical rule Y = X − 3, which correlates the position of the implant from the bone crest level (Y) with the thickness of the soft tissues (X). All the implants were inserted in fresh extraction sockets, and immediately loaded with temporary abutments and prostheses. Bone levels were evaluated through radiographic examination just after surgical procedure (T0), and after 10 days (10D), 6 months (6M), 1 year (1Y), and 5 years (5Y). After 5 years, the implant survival rate was 100%, with a medium marginal bone loss around implants of 0.0704 mm (SD = 0.169 mm). One-way ANOVA, followed by Tukey’s multiple comparison test was performed for statistical evaluations (*p* < 0.05). This protocol provided a safe and successful procedure, with a good soft tissue seal against bacterial challenge. The application of the mathematical rule allows the implant placement in a correct vertical position from the bone crest, avoiding bone resorption and bacterial infiltrations. Moreover, the use of Multi Unit Abutment (MUA) determined a stable biological seal, favouring the implant healing and preserving the adhesion of hemidesmosomes to the titanium of MUA.

## 1. Introduction

In the last decades, modern dentistry observed a rapid evolution of techniques. Implant-rehabilitation protocols have been redefined over the years, as a consequence of continuous research and new materials in implant surgery, increasing patient’s expectations in terms of comfort, aesthetic and reducing treatment periods [1]. Updates protocols have arisen shortening the healing period, so that implants can be loaded early or, even, immediately, before osseointegration is completely achieved. The immediate loading after tooth extraction was introduced in 1976 as an alternative to the classic delayed implant placement described by Branemark [2], and it is widely described in literature [3,4]. This procedure seems to preserve the integrity of the socket itself [5], providing favorable results with outcomes similar to implants placed in healed edentulous ridge [6].

Implants failure is usually related to the development of peri-implantitis associated with bone loss around the implant [7]. A current major challenge is to minimize the marginal bone loss (MBL) around implants, since it has been proven to be important for soft tissue stability and long-term success, as the resorption can trigger bacterial colonization of the coronary part of the implant [8,9,10]. Studies in literature suggested that MBL is influenced by the implant location with respect to the bone crest, the amount of keratinized mucosa and the soft tissue thickness [11,12,13,14].

The subcrestal placement of dental implants may avoid exposure of implant threads after initial physiologic bone remodeling and allow an adequate aesthetic emergence profile for the prosthetic restoration, with a positive impact on the papillae formation [10,12]. 

MBL was firstly described by Berglundh in 1996 [15]. Authors showed that when the flap had a thin connective layer before screwing the healing abutment, there was a proportional bone resorption up to get a space, defined “biological width”, equal to 3 mm, divided into 2 mm of epithelium and 1 mm of connective. Therefore, MBL is correlated to the facial soft tissue level, described by Barone et al. [16,17]. Moreover, the influence of mucosal thickness upon MBL around implant necks was discussed by Cochran et al. (1997) [18], who suggested that soft tissues create a protective barrier against inflammatory infiltration toward the underlying alveolar bone. Further studies evidenced that, to avoid MBL, the vertical mucosal thickness necessary for biological width establishment around implants should be at least 2 mm [19,20]. Linkevicius et al., 2010 reported that after one year loading, implants with an initial vertical mucosal thickness greater than 2 mm maintained marginal bone levels more successfully than implants with a thinner one [21]. More recently, Vervaeke et al., 2014 suggested of adapting the vertical position of the implant to the initial soft tissue thickness in order to avoid unforeseen exposure of the implant surface due to initial bone remodeling; a limitation of this study was the lack of accurate soft tissue measurements [22].

Since soft tissues provide a defensive role [23,24], another factor that can influence MBL is the frequency of exchange of abutment that may disturb the surrounding peri-implant tissues [25]. Indeed, titanium is the most recommended material, since it allows to establish stable hemidesmosomal junctions between the implant and the alveolar bone [26]. To avoid the detachment of these junctions, the number of unscrewing of the healing abutment, before its final insertion, should be reduced to zero. Furthermore, concerning the type of connection, screwed prostheses seem to give more guarantees if compared to cemented ones, both in terms of stability and damages due to the invasion and infiltration of the cement among the biological soft tissue spaces [27,28,29].

Since the main goal of implant success is to prevent bacterial leakage, clinicians should use surgical techniques that minimize the inflammation of soft and hard tissues and avoid bone loss around implant [30]. Up to date, in literature, there are confused information on how many millimeters implants should be placed under the bone crest in order to prevent bacterial infiltration and to prevent MBL. In this light, the purposes of the present study were (1) to scientifically validate a new mathematical rule based on the thickness of soft tissues, to choose the correct implant position with respect to the bone crest, in order to provide a better tissue adaptation to the abutment/implant surface against bacterial invasion, and (2) to apply the mathematical rule to the surgical protocol for implant immediate loading, called Biological Oriented Immediate Loading (B.O.I.L.), to avoid peri-implant bone resorptions and implant failures.

## 2. Materials and Methods

### 2.1. Patients Enrollment

A total of 37 patients (20 women and 17 men, aged 45 to 70 years at time of enrollment) were enrolled in this study. Patients were submitted to surgical procedure with therapeutic purposes; all of them needed extraction of compromised elements and refused to wear a temporary removable prosthesis. All patients were provided oral and written information regarding the surgery and the present study, and they signed an informed consent to participate.

Inclusion criteria in the study were: (i) 18 to 70 years of age; (ii) adequate residual bone quality after extraction, to allow the placement of implants with the minimum diameter of 4 mm and the minimum length of 11.5 mm; (iii) patient’s ability to follow the protocol of the study. Exclusion criteria were: (i) patients with compromised general health conditions; (ii) radiotherapy patients; (iii) pre-existing and ongoing of diphosphonate therapy; (iv) excessive inter-maxillary discrepancies; (v) sever parafunctional habits; (vi) poor oral hygiene; (vii) more than 10 cigarettes per day smokers; (viii) drug or alcohol abuse, and (ix) implants with an implant stability quotient (ISQ) < 60.

Before surgery, patients underwent clinical and imaging examination, by panoramic evaluation (Sidexis Digital System, Dentsply Sirona, York, PA, USA) and digital intraoral periapical radiographs. In 21 patients, a single implant was inserted after tooth extraction, while the other 16 patients received multiple implants for upper or lower full-arch prostheses, for a total of 127 implants.

### 2.2. Development of a Mathematical Rule for Vertical Implant Position

Based on the study of Berglundh et al. [15] and other studies in literature, which consider the influence of soft tissue thickness and biological width on vertical implant position [31], the following mathematical rule was developed, in order to scientifically define the implant placement with respect to the bone crest:

Y = X − K = X − 3(1)

Y was the distance from the bone level to the most coronal part of the implant, X the thickness of the soft tissue and K was a constant related to the biological width, whose value was fixed at 3.

This formula highlights that to obtain a bone resorption around implant equal to zero, the implant vertical position should be decided following the thickness of the soft tissues, in order to anticipate the biological width re-establishment. For example, if the thickness of the soft tissue was 2 mm (X), Y should be −1: this means that the implant should be placed 1 mm under the bone crest level.

### 2.3. Surgical Protocol: “Biological Oriented Immediate Loading” (B.O.I.L)

A total of 127 implants were inserted, following the Biological Oriented Immediate Loading protocol (B.O.I.L). Implant surgery was performed by the same surgeon and all the patients underwent the same protocol. First, they were subjected to oral hygiene in order to lower the bacterial charge and favour the healing of the implants. Before starting the surgical phase, the facial soft tissue level was measured. The thickness of the soft tissues (expressed in millimetres and representing the X value in the mathematical rule) was measured as the distance between the extreme buccal point of the papilla and the radiopacity of bone crest level by intraoral digital radiographic evaluation (Sidexis Digital System, Dentsply Sirona): a guiding system was adopted to obtain the x-rays perpendicular to the film. In case of edentulous areas near the hopeless tooth, this parameter was obtained by calculating the distance between the extreme limit of radio-transparency of the soft tissues and the radio-opacity of the bone crest.

One hour before surgery, patients received 2 g of amoxicillin, and then, just before surgery, 0.2% chlorhexidine mouthwash was performed for 2 min. Patients were subjected to local anaesthesia with Articain, 40 mg/mL 1:100,000, administered near the nerve that innervates the affected area, and Articain, 40 mg/mL 1:1200 administered by infiltration in the areas adjacent to the implants.

The low dose of adrenaline was necessary to reduce local vasoconstriction and encourage modest bleeding and vascular support, to promote healing [32]. Compromised and hopeless teeth were carefully extracted with extraction forceps, trying to minimize trauma to the surrounding bone. The tooth socket was carefully debrided to remove granulation tissue. Special attention was paid to avoid damaging to the soft tissues, using a flapless procedure. Subsequently, Biomet 3i conical implants (diameter 4 mm and minimal length 11.5 mm, Biomax S.p.A, Vicenza, Italy) were inserted into the fresh extraction sites, following the new implant insertion protocol Biological Oriented Immediate Loading (B.O.I.L). According to this, the vertical position of each implant was set using the mathematical rule Y = X − 3, considering the thickness of the soft tissue and at least 3 mm space for the biologic width.

Implant stability was evaluated with Osstel Device System (Integration Diagnostic, Gothenburg, Sweden). Implants with an implant stability quotient (ISQ) < 60, were excluded from this study, following the scientific literature which suggested that, to achieve a good implant primary stability, an ISQ value of more than 60 should be chosen as the minimum value for immediate loading [10,33].

To obtain a good primary stability, it was also used a torque of implant’s insertion of at least 30 N/cm (between 30 and 80 N/cm), value that allows to avoid micro movements that could be critical to bone healing [33]. An excessive value of the torque’s insertion can cause a dangerous deformation of the anti-rotational geometric systems, that fits the abutment, leading to a consequent impairment of the future prosthesis’ stability [34,35].

A low profile Multi Unit Abutment (MUA) (Biomax 3i, Biomax S.p.A, Vicenza, Italy), single-piece or 2-piece types in case of individual elements (ILPC342U or ILPC342), was contextual positioned. A periapical radiograph was taken immediately after the implant surgery in order to determine the starting bone level (T0).

To all patients were prescribed the following medications: amoxicillin (1 g every 12 h for the following 4 days) and anti-inflammatory therapy (1 tablet every 12 h for the following 3 days).

### 2.4. Prosthetic Protocol

Only implants with both torque of implant insertion of 30 N/cm and ISQ > 60 were immediately loaded. A screw-retained transfer for low profile was connected to the implant. Using a polyether rubber material, the impression was immediately taken; to get more detail accuracy, first the heavy component was used and then the light one. Hence, a temporary composite reinforced prosthesis, without occlusal contacts, was prepared and screwed on the Low Profile MUA, within 12 h, using gold retaining screws. A temporary cement (CavitTM 3M ESPE, Saint Paul, MN, USA) was put in the holes of the prosthesis in correspondence with the retaining screws. After 3 days, it was partially removed and the holes were closed, using a resin composite material. After 60 days, the temporary prosthesis was unscrewed and an impression, using polyether rubber material, was taken, in order to display the soft tissues’ state. Finally, the final metal-composite prosthesis was screwed within 90 days from the surgery and never removed. Holes, in correspondence of the screws, were closed using CavitTM (3M ESPE) and resin composite material.

### 2.5. Radiographic Measurements and Evaluation

Clinical examinations were performed just after the surgical procedure (T0), and after 10 days (10D), 6 months (6M), 1 year (1Y), and 5 years (5Y). During this period, patients were enrolled in the maintenance program, which included daily oral hygiene and prophylaxis.

At the same time points, digital intraoral periapical radiographs of each implant were collected (Sidexis Digital System, Dentsply Sirona), using a plastic positioner, according to the parallelism technique. The thickness of soft tissues (X values) was measured at T0, according to the above descripted method. The Bone Level (BL), defined as the distance from the implant neck to the bone crest, was calculated at each time point [36]. The Marginal Bone Loss (MBL) was obtained by subtracting the BL values at T0 to those at 10D, 6M, 1Y and 5Y. Subsequently, MBL data were converted by exploiting a base 10 exponential data transformation, in order to deal with the numerous zero values obtained by the analysis. To this aim, the exact values of MBLwere used as exponential and 10 was kept as base, which led to a normally distributed range of values from 0 to 1.

### 2.6. Statistical Analysis

Data were presented as mean ± standard deviation (SD). Significant differences between groups were determined by means of one-way ANOVA, followed by Tukey’s multiple comparison test, using the statistical software package Prism6 (GraphPad Software 4.0, Inc., San Diego, CA, USA). The level of significance was set at *p* < 0.05. Correlations between soft tissue thickness and marginal bone loss (MBL) around the inserted implants were analyzed using the intra class correlation coefficient. Correlations between MBL around implants subcrestal and equicrestal placed, following the mathematical rule, were analyzed using the intra class correlation coefficient.

## 3. Results

**Figure 1:**
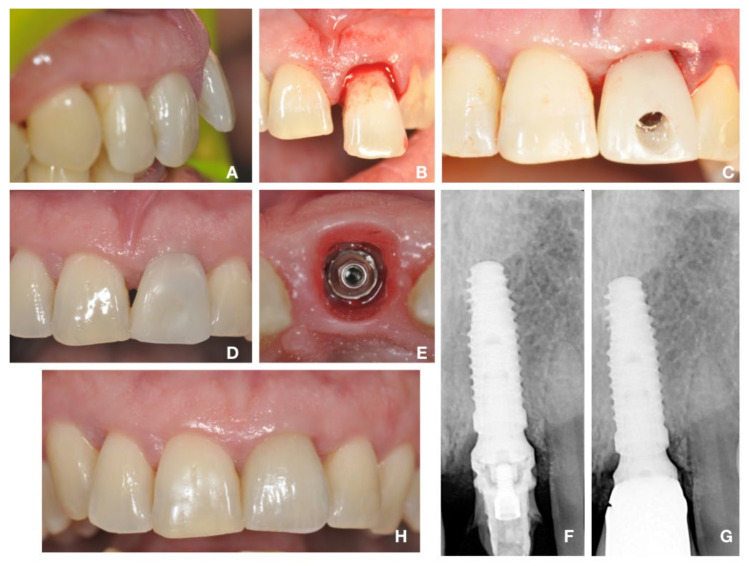
Single restoration in fresh extraction socket, immediately loaded with temporary abutment and prosthesis. (**A**) Clinical situation before the extraction of tooth 2.1. (**B**) Extraction of the hopeless tooth 2.1. (**C**) Implant insertion under the bone crest level, according to the mathematical rule (a low profile MUA was contextually positioned and immediately loaded with a temporary crown). Clinical evaluation after 60 days, showing excellent soft tissue healing around the temporary crown (**D**) and the implant (**E**). Radiographic evaluation after 60 days (**F**) and after 5 years follow-up (**G**), showing no bone resorption around implant. (**H**) Clinical evaluation after 5 years follow-up.

**Figure 2:**
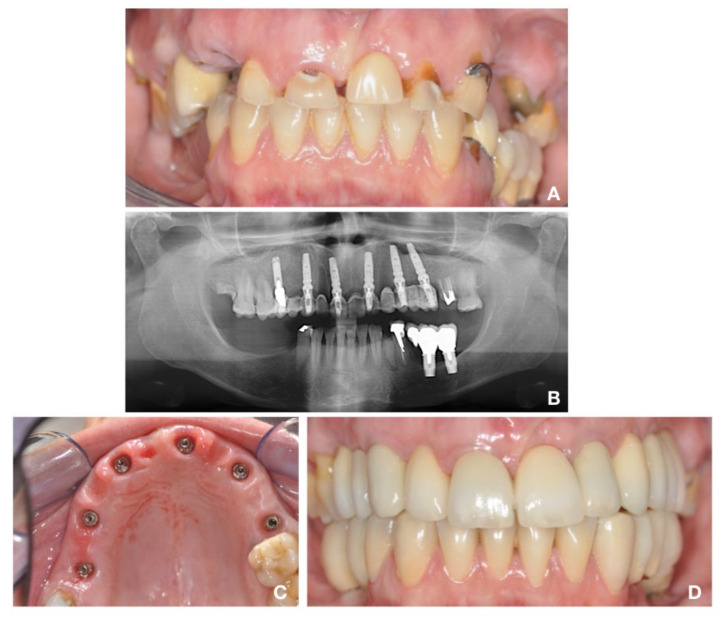
Full arch restoration in fresh extraction sockets, immediately loaded with temporary abutment and prosthesis. (**A**) Clinical situation before teeth extraction. (**B**) Radiographic and (**C**) clinical evaluation after 10 days from implants insertion: soft tissues around the implants did not show signs of inflammation. (**D**) Clinical evaluation after 5 years follow-up: interdental papillae appeared well preserved and no soft tissues retraction occurred.

The clinical and prosthetic protocols of two patients, representative of single and full arch implants, are displayed in Figure 1 and Figure 2. The average time of observation was 60 months. No patients dropped out or were excluded during the follow-up time, and all patients returned for the scheduled follow-up evaluations. No implants or prostheses were rejected within the 60-month evaluation period, resulting in 100% implants and prostheses survival rate. Neither loosening of the prosthetic retention screws nor fractures were recorded. One case experienced the loss of the incisal edge of 1.3; however, it was repaired with composite material without removing the prosthesis. The quality of the soft keratinized tissue was improved after 6 months and remained stable over time for almost all cases.

**Figure 3:**
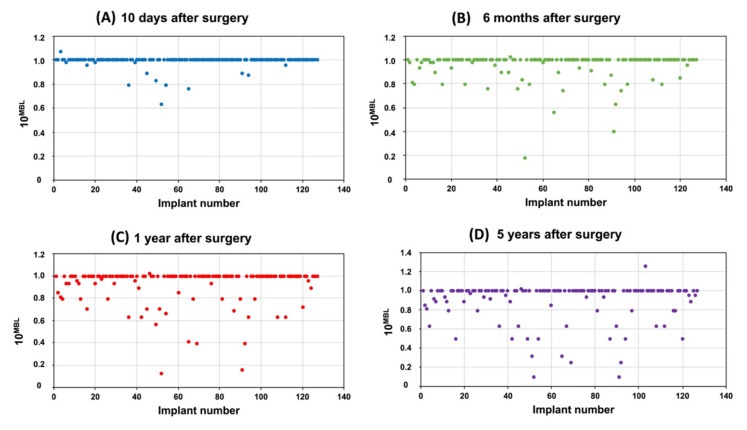
Marginal bone loss (MBL) values calculated around inserted implants at 10 days (**A**), 6 months (**B**), 1 year (**C**), 5 years (**D**) after surgery (MBL values were submitted to a base 10 exponential data transformation, to deal with the numerous zero values).

The MBL values of the analysed implants, calculated at different time points (10D, 6M, 1Y and 5Y follow-up), are reported in Figure 3, by exploiting a base 10 exponential data transformation, as above described: hence, values minor to 1 corresponded to negative MBL (bone resorption), while values equal to 1 to MBL equal to zero (no bone resorption). After 10 days, only 13 implants (10.2%) showed negative MBL values (10^MBL^ values minor to 1), with a mean MBL of 0.0063 mm (Figure 3A). After 6 months, the number of implants with negative MBL was 36 (28.3%), with a medium MBL of 0.0284 mm (Figure 3B). Similar values were observed after 1 and 5 years, with respectively 39 (30.7%; mean MBL 0.0499 mm; Figure 3C) and 43 (33.8%; mean MBL 0.0704 mm; Figure 3D).

**Figure 4:**
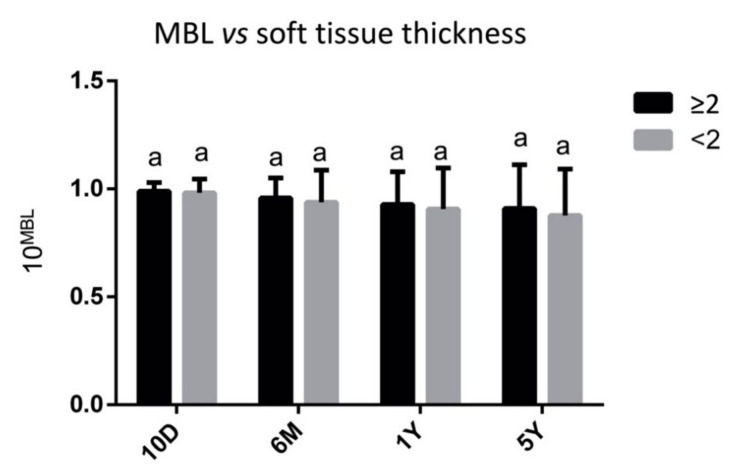
Marginal bone loss (MBL) calculated at 10 days (10D), 6 months (6M), 1 year (1Y) and 5 years (5Y) after surgical procedure, in relation with different soft tissues thickness (≥2 mm and <2 mm). Same letters indicate not statistically significant differences among groups (*p* > 0.05). MBL values were submitted to a base 10 exponential data transformation, to deal with the numerous zero values.

No statistically significant differences (*p* > 0.05) were found in MBL values from implants inserted in patients with a thicker mucosa (≥2 mm) than in patients with a thinner one (<2 mm), both at the same time point and by comparing different times points (Figure 4).

**Figure 5:**
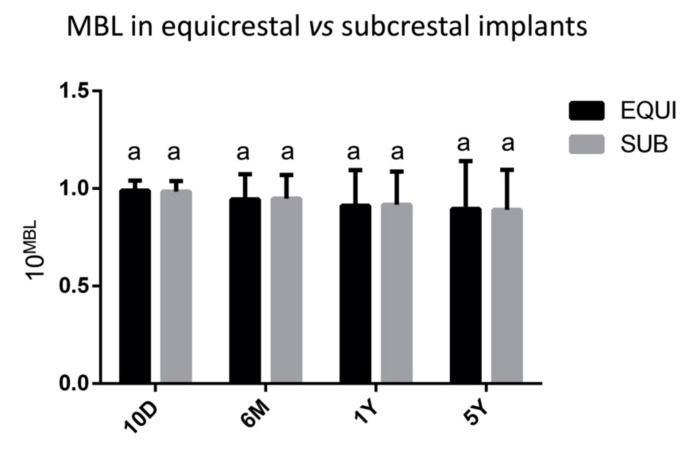
Marginal bone loss (MBL) calculated at 10 days (10D), 6 months (6M), 1 year (1Y) and 5 years (5Y) after surgical procedures, in equi- and subcrestal implants. Same letters indicate no statistically significant differences among groups (*p* > 0.05). MBL values were submitted to a base 10 exponential data transformation, to deal with the numerous zero values.

Following the above described mathematical rule, N. 22 implants were inserted in equicrestal position, N. 5 supracrestal, and N. 100 subcrestal. In the case of supracrestal implants, only one showed, after 5 years, an MBL different from 0 (0.05 mm). Regarding subcrestal and equicrestal implants, they showed similar progressive bone changes at each time point, with no statistical differences (*p* > 0.05) (Figure 5).

## 4. Discussion

The long-term stability of implants as well as the influence of peri-implant mucosal thickness on bone remodeling are currently a topic of debate and enhanced research [4,14]. Likewise natural teeth, dental implants are surrounded by the same soft tissue barrier consisting of an epithelial part and a connective tissue one [22]. Hence, the stability of the crestal bone is believed to be the key factor for maintaining stable soft tissue dimensions over time.

To the best of the authors’ knowledge, there are not clinical studies reporting a mathematical rule based on the thickness of the soft tissue, which allows to calculate the right implant vertical position with respect to the bone crest, in order to avoid bone resorption and bacterial infiltration. As regards this issue, the existing literature is not yet conclusive. Some authors recommended to place the implant under the bone crest, in order to obtain a better emergence profile and prevent the implant surface from being exposed [37,38]. Conversely, other authors reported an increase in bone loss for subcrestal placed implants, due to the low concentration of oxygen that promotes the bacterial colonization on the implant-abutment junction [39,40]. Dibart et al. described that implants placed at the crestal bone level approximated the microgap to the bone and thus MBL would occur due to possible bacterial leakage [41]. Moreover, other studies highlighted that there were not statistically significant differences in bone loss between subcrestal and equicrestal implants, and that the loss of bone was not strictly due to the location of the implant with respect to the bone [10,31,42].

A recent study concluded that the initial bone remodeling is due to the soft tissue thickness, instead of the vertical implant position [12]. At this regard, there is a classification which divided soft tissue into thin (<2 mm) or thick (≥2 mm) with respect to their thikness [43]. A recent systematic review reported that up to date, there is no sufficient evidence of a causal effect between thin soft tissues and crestal bone loss around dental implants [44]. On the other hand, another systematic review, including meta-analysis, concluded that the thickness of soft tissues is essential in terms of initial crestal bone maintenance [20]: implants placed with an initially thicker peri-implant soft tissue showed less radiographic MBL in the short term. Perussola et al. concluded that implants with a keratinized mucosa (KM) < 2 mm exhibited more MBL than sites with keratinized mucosa > 2 mm [13]. Meta-analyses from recent systematic reviews reported significant differences in plaque control, inflammation and bacterial infiltration, suggesting that KM < 2 mm was related with more plaque accumulation and peri-implant tissue inflammation [45,46].

Thus, it appears that soft tissue thickness plays a more important role in MBL and bacterial losses than the vertical position of the implants relative to the bone. Moreover, another factor that affect MBL around dental implant is the biological width [31], analysed also by Vervaeke et al., 2014, 2018 [12,22]. In these studies, the thickness of the soft tissue was measured before placing the implants and the subcrestal implant position was adapted, leaving at least 3 mm of space for the establishment of the biological width. If a patient was characterized by mucosal width of 2 mm, the implant was placed 1 mm below the crest, in order to anticipate bone remodeling and avoid future exposure of the implant.

The present study, in agreement with Vervaeke et al., 2018, scientifically validates a mathematical rule for the vertical position of the implant that takes into account the thickness of the soft tissue and the biological width, in order to facilitate and standardize the clinical practice. Based on this mathematical rule, a new surgical protocol was introduced, named Biological Oriented Immediate Loading (B.O.I.L.). According to the scientific literature, an average biological width of ca. 3 mm is described around non-submerged implants installed in a one-stage surgical procedure [18] and 3.42–3.80 mm around submerged implants installed in a two-stage surgical procedure [47]. According to the study of Berglundh [15], no bone resorption occurs when implants are inserted at the bone crest level and the thickness of the soft tissue is equal to 3 mm. Starting from this result, the mathematical rule, presented in this study, allows to anticipate the biologic width re-establishment, described in literature, adapting the vertical position of the implant, in order to avoid implant surface exposure. Moreover, for this reason, in the present study, there was no statistical differences (*p* > 0.05) between implants placed in patients with thin mucosa compared to patients with thick one.

In the study of Vervaeke et al., 2018, authors reported that the only difference between submerged and non-submerged conditions is the moment when biologic width formation takes place, being after implant placement for non-submerged implants and after the second stage surgery for submerged ones. According to our results, no statistically significant differences were found between equi- and subcrestal implants, since the biological width around implant is calculated before the implant insertion. Thus, this protocol avoids and reduces bone remodeling around implants since after 5 years follow-up. Moreover, following B.O.I.L protocol, the mean MBL values after 1 and 5 years were respectively 0.0499 mm and 0.0704 mm. After five years follow-up, only two cases showed a bone resorption equal to 1 mm, while no bone resorption was observed in 81 implants. Hence, this protocol allows to avoid after 5 years follow-up the formation of a gap and the implant exposure, preventing bacterial infiltration. These data are lower than those reported in literature. In fact, in 1986, Albrektsson et al., published a paper, which is still a reference today, where it is argued that a bone loss less than 1.5 mm during the first year after implant placement and less of 0.2 mm annually in the following years, can be considered satisfactory [48]. Furthermore, in a recent retrospective cohort study, authors reported that the MBL around implants of various designs was 0.06 ± 0.22 mm at 3 months and it minimally increased to 0.44 ± 0.81 after 10 years [49].

The simultaneous insertion of MUA allows the adhesion of hemidesmosomes around the titanium with the formation of a stable biological seal. It allows to create a coagulum chamber, described by Degidi et al., which is essential for the proper healing of the socket with the consequent reduction of bone resorption [50]. Furthermore, since the bacterial leakage in the implant-abutment interface is raised as the most important factor in the occurrence of inflammatory reactions around the implant [51,52], B.O.I.L protocol allows to not unscrew the healing abutment, before its final insertion, so as to prevent bacterial infiltration and the detachment of the hemidesmosomial junctions.

These findings are based on a limited sample size. Hence, future studies with a larger cohort of patients are needed to elucidate this topic, also comparing implants placed according to the mathematical rule with implants inserted according to the manufacturer’s guidelines (equicrestal placement). Within the limitations of this study, it can be concluded that the advantages of B.O.I.L. protocol are the good aesthetic results, excellent soft tissue healing, with a stable mucogingival junction and the preservation of the interproximal papillae. Moreover, this surgical protocol allows to insert implants in a correct position with respect to the bone crest, according to the soft tissues thickness, avoiding marginal bone resorption due to bacterial infiltration and obtaining a better healing, thanks to the use of MUA that allows to create a coagulum chamber, ensuring the coagulum’s stability.

## 5. Conclusions

This study leads to a relevant impact on clinical practice, since in literature it is not well defined how many millimetres implants should be submerged under the bone crest, in order to avoid marginal bone loss around dental implants. Early clinical studies had suggested that this bone loss occurred during the first year of loading. Although the reason and the time for this bone loss are still unknown, numerous attempts were made to minimize or eliminate it. One hypothesis was related to the presence of bacteria in the interfaces between the implant and abutment connections [53,54].

The obtained results suggested that B.O.I.L. protocol can help implants insertion, in term of choosing the correct implant vertical position, based on the thickness of the soft tissues. Moreover, this protocol allows to avoid bacterial infiltration and the consequent peri-implant bone resorption. Early implant surface exposure may increase the risk for peri-implantitis; hence, placing implant with respect to soft tissue thickness is advised.

In conclusion; B.O.I.L. protocol can be considered a reliable approach for implant supported fixed prostheses. The mathematical rule let customize and simplify the surgery, allowing to obtain excellent results after 5 years follow-up. Additional studies with a larger number of patients are warranted to reinforce the obtained findings and verify the mathematical rule.

## Data Availability

Data available on request due to restrictions eg privacy. The data presented in this study are available on request from the corresponding author.

## References

[1] Tettamanti L., Andrisani C., Bassi M.A., Vinci R., Silvestre-Rangil J., Tagliabue A. (2017). Immediate Loading Implants: Review of the Critical Aspects. Oral Implantol..

[2] Schulte W., Heimke G. (1976). The Tübinger immediate implant. Quintessenz.

[3] Chiapasco M., Gatti C. (2003). Implant-Retained Mandibular Overdentures with Immediate Loading: A 3- to 8-Year Prospective Study on 328 Implants. Clin. Implant. Dent. Relat. Res..

[4] Esposito M., Grusovin M.G., Maghaireh H., Worthington H.V. (2013). Interventions for Replacing Missing Teeth: Different Times for Loading Dental Implants. Cochrane Database Syst. Rev..

[5] Degidi M., Piattelli A., Iezzi G., Carinci F. (2007). Retrospective Study of 200 Immediately Loaded Implants Retaining 50 Mandibular Overdentures. Quintessence Int..

[6] Ragucci G.M., Elnayef B., Criado-Cámara E., Del Amo F.S.-L., Hernández-Alfaro F. (2020). Immediate Implant Placement in Molar Extraction Sockets: A Systematic Review and Meta-Analysis. Int. J. Implant. Dent..

[7] Ragucci G.M., Giralt-Hernando M., Méndez-Manjón I., Cantó-Navés O., Hernández-Alfaro F. (2020). Factors Affecting Implant Failure and Marginal Bone Loss of Implants Placed by Post-Graduate Students: A 1-Year Prospective Cohort Study. Materials.

[8] Aslam A., Hassan S.H., Aslam H.M., Khan D.A. (2019). Effect of Platform Switching on Peri-Implant Bone: A 3D Finite Element Analysis. J. Prosthet. Dent..

[9] Spinato S., Stacchi C., Lombardi T., Bernardello F., Messina M., Zaffe D. (2019). Biological Width Establishment around Dental Implants Is Influenced by Abutment Height Irrespective of Vertical Mucosal Thickness: A Cluster Randomized Controlled Trial. Clin. Oral Implant. Res..

[10] De Siqueira R.A.C., Gonçalves R.S., Dos Santos P.G.F., de Mattias Sartori I.A., Wang H.-L., Fontão F.N.G.K. (2020). Effect of Different Implant Placement Depths on Crestal Bone Levels and Soft Tissue Behavior: A 5-Year Randomized Clinical Trial. Clin. Oral Implant. Res..

[11] Van Eekeren P., van Elsas P., Tahmaseb A., Wismeijer D. (2017). The Influence of Initial Mucosal Thickness on Crestal Bone Change in Similar Macrogeometrical Implants: A Prospective Randomized Clinical Trial. Clin. Oral Implant. Res..

[12] Vervaeke S., Matthys C., Nassar R., Christiaens V., Cosyn J., De Bruyn H. (2018). Adapting the Vertical Position of Implants with a Conical Connection in Relation to Soft Tissue Thickness Prevents Early Implant Surface Exposure: A 2-Year Prospective Intra-Subject Comparison. J. Clin. Periodontol..

[13] Perussolo J., Souza A.B., Matarazzo F., Oliveira R.P., Araújo M.G. (2018). Influence of the Keratinized Mucosa on the Stability of Peri-Implant Tissues and Brushing Discomfort: A 4-Year Follow-up Study. Clin. Oral Implant. Res..

[14] Cochran D.L., Nummikoski P.V., Schoolfield J.D., Jones A.A., Oates T.W. (2009). A Prospective Multicenter 5-Year Radiographic Evaluation of Crestal Bone Levels over Time in 596 Dental Implants Placed in 192 Patients. J. Periodontol..

[15] Berglundh T., Lindhe J. (1996). Dimension of the Periimplant Mucosa. Biological Width Revisited. J. Clin. Periodontol..

[16] Barone A., Toti P., Quaranta A., Derchi G., Covani U. (2015). The Clinical Outcomes of Immediate Versus Delayed Restoration Procedures on Immediate Implants: A Comparative Cohort Study for Single-Tooth Replacement. Clin. Implant. Dent. Relat. Res..

[17] Barone A., Toti P., Piattelli A., Iezzi G., Derchi G., Covani U. (2014). Extraction Socket Healing in Humans after Ridge Preservation Techniques: Comparison between Flapless and Flapped Procedures in a Randomized Clinical Trial. J. Periodontol..

[18] Cochran D.L., Hermann J.S., Schenk R.K., Higginbottom F.L., Buser D. (1997). Biologic Width around Titanium Implants. A Histometric Analysis of the Implanto-Gingival Junction around Unloaded and Loaded Nonsubmerged Implants in the Canine Mandible. J. Periodontol..

[19] Linkevicius T., Apse P., Grybauskas S., Puisys A. (2009). The Influence of Soft Tissue Thickness on Crestal Bone Changes around Implants: A 1-Year Prospective Controlled Clinical Trial. Int. J. Oral Maxillofac. Implants.

[20] Suárez-López Del Amo F., Lin G.-H., Monje A., Galindo-Moreno P., Wang H.-L. (2016). Influence of Soft Tissue Thickness on Peri-Implant Marginal Bone Loss: A Systematic Review and Meta-Analysis. J. Periodontol..

[21] Linkevicius T., Apse P., Grybauskas S., Puisys A. (2010). Influence of Thin Mucosal Tissues on Crestal Bone Stability around Implants with Platform Switching: A 1-Year Pilot Study. J. Oral Maxillofac. Surg..

[22] Vervaeke S., Dierens M., Besseler J., De Bruyn H. (2014). The Influence of Initial Soft Tissue Thickness on Peri-Implant Bone Remodeling. Clin. Implant. Dent. Relat. Res..

[23] Kawahara H., Kawahara D., Hashimoto K., Takashima Y., Ong J.L. (1998). Morphologic Studies on the Biologic Seal of Titanium Dental Implants. Report I. In Vitro Study on the Epithelialization Mechanism around the Dental Implant. Int. J. Oral Maxillofac. Implants.

[24] Kawahara H., Kawahara D., Mimura Y., Takashima Y., Ong J.L. (1998). Morphologic Studies on the Biologic Seal of Titanium Dental Implants. Report II. In Vivo Study on the Defending Mechanism of Epithelial Adhesions/Attachment against Invasive Factors. Int. J. Oral Maxillofac. Implants.

[25] Rompen E. (2012). The Impact of the Type and Configuration of Abutments and Their (Repeated) Removal on the Attachment Level and Marginal Bone. Eur. J. Oral Implantol..

[26] Briguglio F., Falcomatà D., Marconcini S., Fiorillo L., Briguglio R., Farronato D. (2019). The Use of Titanium Mesh in Guided Bone Regeneration: A Systematic Review. Int. J. Dent..

[27] Lang N.P., Berglundh T., Heitz-Mayfield L.J., Pjetursson B.E., Salvi G.E., Sanz M. (2004). Consensus Statements and Recommended Clinical Procedures Regarding Implant Survival and Complications. Int. J. Oral Maxillofac. Implants.

[28] Linkevicius T., Apse P. (2008). Influence of Abutment Material on Stability of Peri-Implant Tissues: A Systematic Review. Int. J. Oral Maxillofac. Implants.

[29] Lauer G., Wiedmann-Al-Ahmad M., Otten J.E., Hübner U., Schmelzeisen R., Schilli W. (2001). The Titanium Surface Texture Effects Adherence and Growth of Human Gingival Keratinocytes and Human Maxillar Osteoblast-like Cells in Vitro. Biomaterials.

[30] Carinci F., Lauritano D., Cura F., Lopez M.A., Andreasi Bassi M., Confalone L., Pezzetti F. (2016). Prevention of Bacterial Leakage at Implant-Abutment Connection Level: An in Vitro Study of the Efficacy of Three Different Implant Systems. J. Biol. Regul. Homeost. Agents.

[31] Palacios-Garzón N., Velasco-Ortega E., López-López J. (2019). Bone Loss in Implants Placed at Subcrestal and Crestal Level: A Systematic Review and Meta-Analysis. Materials.

[32] Libonati A., Nardi R., Gallusi G., Angotti V., Caruso S., Coniglione F., Marzo G., Mattei A., Tecco S., Paglia L. (2018). Pain and Anxiety Associated with Computer-Controlled Local Anaesthesia: Systematic Review and Meta-Analysis of Cross-over Studies. Eur. J. Paediatr. Dent..

[33] Papaspyridakos P., Chen C.-J., Chuang S.-K., Weber H.-P. (2014). Implant Loading Protocols for Edentulous Patients with Fixed Prostheses: A Systematic Review and Meta-Analysis. Int. J. Oral Maxillofac. Implants.

[34] Bambini F., Memè L., Pellecchia M., Sabatucci A., Selvaggio R. (2005). Comparative Analysis of Deformation of Two Implant/Abutment Connection Systems during Implant Insertion. An in Vitro Study. Minerva Stomatol..

[35] Marchetti E., Ratta S., Mummolo S., Tecco S., Pecci R., Bedini R., Marzo G. (2014). Evaluation of an Endosseous Oral Implant System According to UNI EN ISO 14801 Fatigue Test Protocol. Implant. Dent..

[36] Peñarrocha M., Palomar M., Sanchis J.M., Guarinos J., Balaguer J. (2004). Radiologic Study of Marginal Bone Loss around 108 Dental Implants and Its Relationship to Smoking, Implant Location, and Morphology. Int. J. Oral Maxillofac. Implants.

[37] Wennerberg A., Sennerby L., Kultje C., Lekholm U. (2003). Some Soft Tissue Characteristics at Implant Abutments with Different Surface Topography. A Study in Humans. J. Clin. Periodontol..

[38] Barros R.R.M., Novaes A.B., Muglia V.A., Iezzi G., Piattelli A. (2010). Influence of Interimplant Distances and Placement Depth on Peri-Implant Bone Remodeling of Adjacent and Immediately Loaded Morse Cone Connection Implants: A Histomorphometric Study in Dogs. Clin. Oral Implant. Res..

[39] Pellicer-Chover H., Peñarrocha-Diago M., Peñarrocha-Oltra D., Gomar-Vercher S., Agustín-Panadero R., Peñarrocha-Diago M. (2016). Impact of Crestal and Subcrestal Implant Placement in Peri-Implant Bone: A Prospective Comparative Study. Med. Oral Patol. Oral Cir. Bucal..

[40] Do Nascimento C., Miani P.K., Pedrazzi V., Muller K., de Albuquerque R.F. (2012). Bacterial Leakage along the Implant-Abutment Interface: Culture and DNA Checkerboard Hybridization Analyses. Clin. Oral Implant. Res..

[41] Dibart S., Warbington M., Su M., Skobe Z. (2005). In Vitro Evaluation of the Implant-Abutment Bacterial Seal: The Locking Taper System. Int. J. Oral Maxillofac. Implants.

[42] De Siqueira R.A.C., Fontão F.N.G.K., Sartori I.A.d.M., Santos P.G.F., Bernardes S.R., Tiossi R. (2017). Effect of Different Implant Placement Depths on Crestal Bone Levels and Soft Tissue Behavior: A Randomized Clinical Trial. Clin. Oral Implant. Res..

[43] Linkevicius T., Puisys A., Steigmann M., Vindasiute E., Linkeviciene L. (2015). Influence of Vertical Soft Tissue Thickness on Crestal Bone Changes Around Implants with Platform Switching: A Comparative Clinical Study. Clin. Implant. Dent. Relat. Res..

[44] Akcalı A., Trullenque-Eriksson A., Sun C., Petrie A., Nibali L., Donos N. (2017). What Is the Effect of Soft Tissue Thickness on Crestal Bone Loss around Dental Implants? A Systematic Review. Clin. Oral Implant. Res..

[45] Gobbato L., Avila-Ortiz G., Sohrabi K., Wang C.-W., Karimbux N. (2013). The Effect of Keratinized Mucosa Width on Peri-Implant Health: A Systematic Review. Int. J. Oral Maxillofac. Implants.

[46] Lin G.-H., Chan H.-L., Wang H.-L. (2013). The Significance of Keratinized Mucosa on Implant Health: A Systematic Review. J. Periodontol..

[47] Bernardi S., Mummolo S., Tecco S., Continenza M.A., Marzo G. (2017). Caracterización Histológica de La Membrana de Los Factores de Crecimiento Concentrados Sacco. Int. J. Morphol..

[48] Albrektsson T., Zarb G., Worthington P., Eriksson A.R. (1986). The Long-Term Efficacy of Currently Used Dental Implants: A Review and Proposed Criteria of Success. Int. J. Oral Maxillofac. Implants.

[49] French D., Cochran D.L., Ofec R. (2016). Retrospective Cohort Study of 4,591 Straumann Implants Placed in 2,060 Patients in Private Practice with up to 10-Year Follow-up: The Relationship Between Crestal Bone Level and Soft Tissue Condition. Int. J. Oral Maxillofac. Implants.

[50] Degidi M., Daprile G., Nardi D., Piattelli A. (2013). Immediate Provisionalization of Implants Placed in Fresh Extraction Sockets Using a Definitive Abutment: The Chamber Concept. Int. J. Periodont. Restor. Dent..

[51] D’Ercole S., Tripodi D., Marzo G., Bernardi S., Continenza M.A., Piattelli A., Iaculli F., Mummolo S. (2015). Microleakage of Bacteria in Different Implant-Abutment Assemblies: An in Vitro Study. J. Appl. Biomater. Funct. Mater..

[52] Lauritano D., Moreo G., Lucchese A., Viganoni C., Limongelli L., Carinci F. (2020). The Impact of Implant–Abutment Connection on Clinical Outcomes and Microbial Colonization: A Narrative Review. Materials.

[53] Sasada Y., Cochran D.L. (2017). Implant-Abutment Connections: A Review of Biologic Consequences and Peri-Implantitis Implications. Int. J. Oral Maxillofac. Implants.

[54] King G.N., Hermann J.S., Schoolfield J.D., Buser D., Cochran D.L. (2002). Influence of the Size of the Microgap on Crestal Bone Levels in Non-Submerged Dental Implants: A Radiographic Study in the Canine Mandible. J. Periodontol..

